# St. John's Hospital, Oxford

**Published:** 1905-07-15

**Authors:** 


### ST. JOHN'S HOSPITAL, OXFORD.

### A HOME OF REST FOR INCURABLES.

No class of charity appeals more keenly to our sympathy than Homes for the Incurable. The arguments which are raised against the partial cure of the unfit do not apply here. The Home for Incurables relieves the bread-winner of what is sometimes almost a paralysing burden, and secures to the afflicted that care, consideration, and peace which it is almost impossible to command in a household of the healthy. Such institutions, therefore, as the Hospital of St. John, at Oxford, should not be allowed to appeal in vain for the necessary support. The home provides for the class which is especially in need of such a refuge—females of the better class and poor ladies. Here they find, often for life, a home of peace within the walls of a charming old house, which is surrounded by a beautiful garden. The institution is under the management of the All Saints' Sisterhood. The accommodation consists of cubicles for the poorer patients, and single rooms for those who can afford to pay a little more. But the fees received are in all cases so small that the institution can never be self-supporting, even under the economical management of the sisters, who not only devote themselves to the care of the inmates, but also assist in a little industry of needlework and cookery to help support the work. They are, however, not content with the work they do—they are eager for more—and appeal very earnestly for funds to extend the accommodation of the home. Where such benefits are to be obtained, there can be no lack of candidates for them, and the present building is quite inadequate to meet the appeals for accommodation. Possibly St. John's Hospital is not as well known as it should be, but we think that the appeal of the good sisters should not remain without a prompt response.

